# The Ionophoric Activity of a Pro-Apoptotic VEGF165 Fragment on HUVEC Cells

**DOI:** 10.3390/ijms21082866

**Published:** 2020-04-20

**Authors:** Stefania Zimbone, Anna M. Santoro, Diego La Mendola, Chiara Giacomelli, Maria L. Trincavelli, Marianna F. Tomasello, Danilo Milardi, Sara García-Viñuales, Michele F. M. Sciacca, Claudia Martini, Giulia Grasso

**Affiliations:** 1 CNR Istituto di Cristallografia Sede Secondaria di Catania, Via Gaifami 18, 95126 Catania, Italy; stefania_zimbone@libero.it (S.Z.); annamaria.santoro@cnr.it (A.M.S.); mariannaflora.tomasello@cnr.it (M.F.T.); danilo.milardi@cnr.it (D.M.); sara.garciavinuales@ibb.cnr.it (S.G.-V.); michele.sciacca@unict.it (M.F.M.S.); 2 Dipartimento di Farmacia, Università di Pisa, Via Bonanno Pisano 6, 56126 Pisa, Italy; lamendola@farm.unipi.it (D.L.M.); chiara.giacomelli@unipi.it (C.G.); maria.trincavelli@unipi.it (M.L.T.); claudia.martini@unipi.it (C.M.)

**Keywords:** copper, peptides, vascular endothelial growth factor, ionophore, HUVEC, apoptosis, membrane model, ESI-MS, angiogenesis

## Abstract

Copper plays an important role as a regulator in many pathologies involving the angiogenesis process. In cancerogenesis, tumor progression, and angiogenic diseases, copper homeostasis is altered. Although many details in the pathways involved are still unknown, some copper-specific ligands have been successfully used as therapeutic agents. Copper-binding peptides able to modulate angiogenesis represent a possible way to value new drugs. We previously reported that a fragment (VEGF73-101) of vascular endothelial growth factor (VEGF165), a potent angiogenic, induced an apoptotic effect on human umbilical vein endothelial cells. The aim of this study was to investigate the putative copper ionophoric activity of VEGF73-101, as well as establish a relationship between the structure of the peptide fragment and the cytotoxic activity in the presence of copper(II) ions. Here, we studied the stoichiometry and the conformation of the VEGF73-101/Cu(II) complexes and some of its mutated peptides by electrospray ionization mass spectrometry and circular dichroism spectroscopy. Furthermore, we evaluated the effect of all peptides in the absence and presence of copper ions by cell viability and cytofuorimetric assays. The obtained results suggest that VEGF73-101 could be considered an interesting candidate in the development of new molecules with ionophoric properties as agents in antiangiogenic therapeutic approaches.

## 1. Introduction

Copper is a crucial player in the angiogenic process as it aids the formation of new blood vessels from pre-existing ones [1]. Angiogenesis sustains tumor and metastasis development [1], and high levels of copper are found in the plasma of patients affected by tumors [2,3,4]. The reasons for this elevation remain unclear, however, the administration of copper sequestering agents as an antitumor approach is under evaluation [5,6]. Recently, a study regarding the evaluation of novel copper-based complexes was conducted. It showed remarkable activities, both in vitro and in vivo, as a potential anti-cancer and anti-angiogenesis copper-containing drug [7].

Copper is required for the activation of the hypoxia-inducible factor-1, a major transcription factor regulating the expression of an essential angiogenic factor, the vascular endothelial growth factor (VEGF) [8,9,10,11,12]. The role of metal in the production of VEGF supports copper’s involvement in blood vessel formation, strengthening the application of copper chelators in anti-angiogenesis therapy [13,14,15]. Furthermore, the angiogenic action of other growth factors, such as basic fibroblastic growth factor (β-FGF), is copper dependent, and the inhibition of angiogenic activity [16,17] can be induced by a decrease in copper concentration. In in vitro experiments, the internalization of copper complexes may involve specific transporters of metal [18,19,20], or occur via passive diffusion. For this reason, the chemical features of different ligands appear to be central factors in the activity of the metal complex because they may influence the metal uptake mechanism [21]. Some of these aspects are the lipophilic character, which impacts cell membrane permeation, the influence on redox state of the metal, the ability to direct the complex to specific cellular targets, and the tendency to exhibit intrinsic cytotoxic activity when dissociated from the metal.

New classes of metal-binding peptides have been synthesized as angiogenesis modulators, by taking inspiration from the natural metal peptides or metalloproteins [22]. In this context, some VEGF mimetic peptides with antiangiogenic activity have been described [23,24,25,26,27].

The vascular endothelial growth factor family (VEGFs) involves polypeptides belonging to the cysteine-knot superfamily of signaling proteins, which includes six structurally related factors in humans: VEGFA, VEGFB, VEGFC, VEGFD, placenta growth factor (PLGF), and platelet-derived growth factor (PDGF). VEGF-A is the most potent and specific regulator of physiological and pathological angiogenesis, and it exists in different isoforms as a result of alternative splicing. VEGF165 (the 165 amino acid isoform of vascular endothelial growth factor, that will be also referred to VEGF in the text) represents the predominant isoform in human tissue and contains a heparin binding domain [28]. It exerts its biological action by binding to the receptor tyrosine kinases VEGF receptor-1 (VEGFR-1) and -2 (VEGFR-2), as well as to the co-receptor neuropilin-1 (NRP-1), all of which are predominantly expressed on endothelial cells. Although VEGF-A ligands bind to both VEGFR1 and VEGFR2, they primarily signal endothelial cell proliferation, survival, migration, and vascular permeability via VEGFR2 [29]. Upon ligand binding, VEGFR-2 undergoes dimerization and strong autophosphorylation of the cytoplasmic domains on specific tyrosine residues, resulting in the subsequent signal propagation [30]. In 1997, the main amino acid residues for VEGF165 binding to VEGFR2 were identified. In particular, Gln 79 and Ile 83, in strands β5-β6, Ile-46 in strand β2, Glu-64 in loop β3- β4, and Phe-17 in helix α1 were deemed pivotal for proper binding. The mutation of each of these key residues to alanine produces the reduction of the VEGF affinity to VEGFR2 by 89, 250, 330, 110, and 90-fold, respectively [31]. These results suggest that it is sufficient to change only specific residues to modify the binding features of VEGFR2 [32,33].

In our recent work, we reported on the pro-apoptotic activity of the VEGF73-101 peptide, which encompasses the β5-β6 loop of the VEGF165 protein sequence [34]. Specifically, VEGF73-101 combines two amino acid sequences with the following dissimilar characteristics: 73–85 sequence rich in hydrophobic residues and involved in binding with VEGFR2, and 85–101 sequence characterized by the presence of three histidines able to coordinate Cu(II) [23,34]. This combinational approach, employing basic folding principles and incorporating short sequences or key residues from the β-sheet domain, was also reported for β-sheet-forming peptide design [35,36]. We showed that the VEGF73-101 peptide induced endothelial cell death and counteracted the proliferative effect of VEGF165 [34]. In addition, the β-sheet conformation of VEGF73-101 suggested that the peptide could adopt a configuration similar to the antiparallel β-sheet structure of the VEGF165 β5-β6 loop. In this paper, we investigated the activity of VEGF73-101, showing that the pro-apoptotic properties of this peptide could be also ascribed to ionophoric activity following its interaction with copper. To establish a relationship between the structure of VEGF73-101 and the cytotoxic activity of the resulting copper(II) complexes, specific structural modifications were introduced to the VEGF73-101 fragment via two singly mutated peptides. Specifically, we introduced two point mutations, in position 79 (Gln-Gly) and 83 (Ile-Gly), close to the metal coordination region, a histidine rich domain, to evaluate if the pro-apoptotic properties and ionophoric activity of the wild type peptide were structure-dependent. The slighter activity of both single mutated peptides indicated that the whole native sequence VEGF73-101 holds pro-apoptotic ability on endothelial cells as a result of conformational, hydrophobic and cell penetrating features. These findings may provide new insights into the design of effective anti-angiogenic therapies.

## 2. Results and Discussion

### 2.1. Peptides: Hydrophobicity and Conformational Analysis

The physicochemical properties of peptides strongly affect the permeability of the cell membrane, and the hydrophobicity is as important as the secondary structure. Therefore, the high-performance liquid chromatography retention times could be related to the overall hydrophobicity of peptides.

To evaluate the importance of the VEGF73-101 peptide structure, we designed two new sequences (VEGFQ79G and VEGFI83G) by replacing two aminoacidic residues in position 79 (Q vs. G) and 83 (I vs. G), respectively. These residues are included in the hydrophobic sequence region of VEGF73-101, and are indicated [34] as crucial for VEGF-VEGFR interactions (for detailed sequences of all peptides included in this study, see Table 1).

Here, we tested peptide lipophilic features by high-performance liquid chromatography using a reverse phase RP18 column [20]. The VEGF73-101 HPLC retention time (t_R_) was 21.7 min (Figure S1), indicating that its hydrophobicity was higher than that of the peptides (Table 2) whose hydrophobicity changed, according to the retention time in the order VEGFQ79G>VEGFI83G>VEGF84-101. As previously suggested [19], the lipophilic character of peptides, combined with their peculiarities, could contribute to modulating the cellular membrane/peptide interactions and their cellular uptake rate.

**Figure 1:**
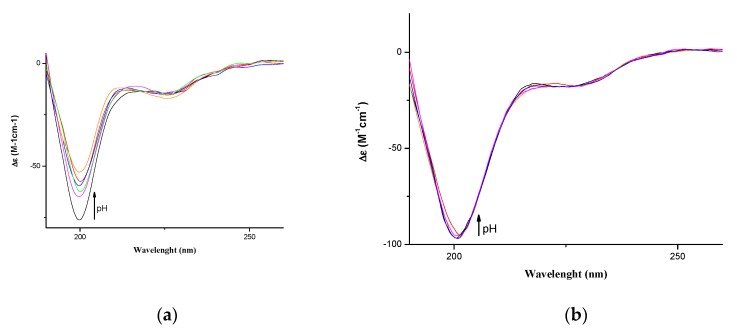
Far-UV CD spectra of (**a**) VEGFQ79G and (**b**) VEGFI83G in water at different pH 5-10 values.

In order to identify the structures responsible for the biological activity of VEGF73-101 we carried out a conformational analysis of VEGFQ79G and VEGFI83G to highlight the differences with the wild type peptide. The Far-UV CD spectra of VEGFQ79G and VEGFI83G in the 5–10 pH range, (Figure 1) showed the presence of a negative band centered at ~200 nm and a very small band centered at 225 nm, indicated a prevalent random coil conformation. These results were similar to those of VEGF84-101 and VEGF84-91 [34] but, at the same time, dissimilar to native sequence VEGF73-101 [34], which exhibited a β-sheet conformation. At any rate, these results indicated that the variations of one amino acid alone can modify the secondary structure of the native peptide, inducing the loss of the β-sheet conformation observed for the wild type peptide [34] and favoring a prevalent random coil structure.

### 2.2. Peptide Permeability on a Membrane Model System

**Figure 2:**
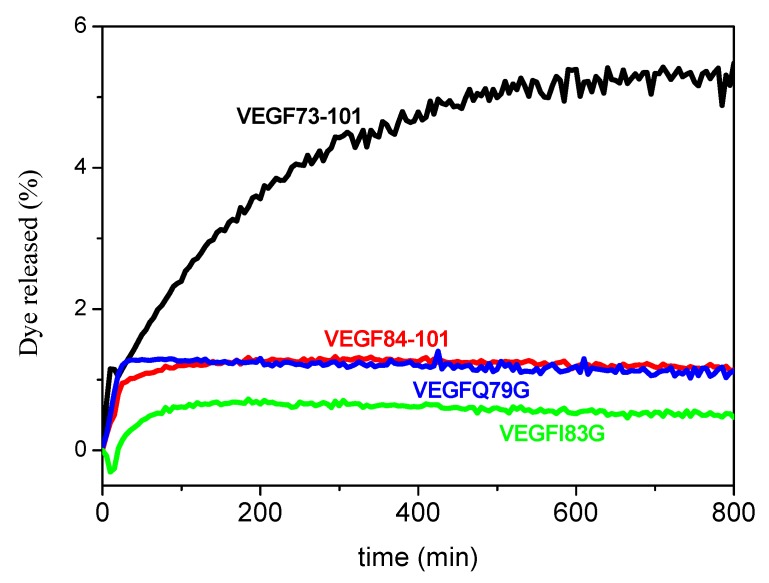
Dye release (membrane leakage) from model membranes induced by VEGF73-101, VEGF84-101, VEGFQ79G, and VEGFI83G. Kinetics of dye release from 200 µM LUVs of 5:3:2 POPC/POPE/POPS in the presence of 10 µM of each peptide. All experiments were performed in 10 mM phosphate buffer, 100 mM NaCl, pH 7.4, at 25 °C. Results are the average of three experiments.

We performed circular dichroism measurements of VEGF fragments in multilamellar vesicles (MLVs) to investigate the peptide’s permeabilization on a membrane model system able to mimic the lipid composition of endothelial cell membranes. The UV-CD of peptides in the membrane model confirmed the conformational differences between VEGF73-101 and the other peptides. In non-aqueous solutions, each peptide, aside from the wild type peptide, showed a random coil profile. In particular, VEGF73-101, upon membrane interactions, changed its conformation from a prevalently β-sheet to a mixture of β-sheet and α-helix secondary structure (Figure S2). We also examined peptide-induced membrane permeation by monitoring the increase in fluorescent emission from the release of 6-carboxyfluorescein from dye-encapsulated vesicles (Figure 2). We observed that only the wild type peptide induced rapid membrane permeabilization that approximated 5% of dye release after 800 min.

Although a thorough description of the membrane permeability mechanism is beyond the aim of this report, we can reasonably ascribe the different membrane permeation of VEGF73-101 to its hydrophobic character and conformation. This behavior reconciles with the membrane-induced conformational transitions of VEGF73-101, showing that alpha-helix structures increase in the presence of LUVs. It is noteworthy to mention that most of membrane-active peptides exhibit a shift toward a higher alpha helical content when added to membranes [37,38,39]. Therefore, peptide conformational properties may steer their ability to permeate the lipid bilayer.

### 2.3. Interaction with Plasma Membrane in Living Cells

**Figure 3:**
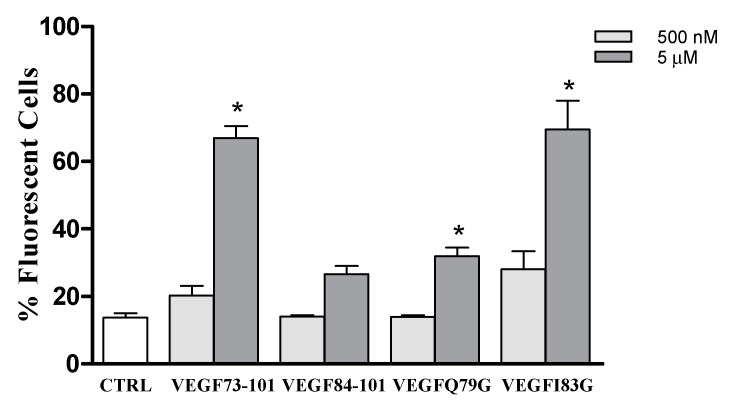
Histograms showing the number of fluorescent cells, expressed as %, of the whole populations treated with the above indicated FAM-conjugated peptides. Data are the mean ± SEM of three different experiments performed in triplicate. Statistically significant differences are indicated with * = *p* < 0.05 vs. CTRL (One-Way ANOVA + Tukey’s test).

Based on the membrane permeability assay reported in Figure 2, VEGF73-101 is the only peptide able to perturb the artificial membrane. To investigate whether or not our peptides can interact with cultured cells, we used 6-carboxyfluorescein (FAM)-conjugated (peptides and flow cytometry analysis. Human umbilical vein endothelial cells (HUVEC)cells were exposed for 24 h to 500 nM or 5 µM of the following peptides: VEGF73-101FAM, VEGF84-101FAM, VEGFQ79GFAM, and VEGFI83GFAM. FAM fluorescence was measured by means of flow cytometry. The percentage of fluorescent cells for each sample is reported in Figure 3. For each sample examined, we found that 5 µM of FAM-conjugated peptide produced fluorescence in the treated HUVEC cells, with the highest percentage obtained for VEGF73-101FAM and VEGFI83GFAM (Figure 3).

Considering that the VEGF73-101FAM was able to perturb membrane model systems, it is conceivable to speculate that HUVEC cells would become fluorescent as a result of peptide internalization or peptide association within membranes. On the other hand, we also found a similar percentage of fluorescent cells in the VEGFI83GFAM sample and, to a lesser extent, in VEGF84-101FAM and VEGFQ79GFAM samples, which did not perturb the membrane model systems. However, membrane model systems are significantly different from the plasma membrane of living cultured cells, both in terms of lipid composition and environmental factors. Furthermore, although flow cytometry allows high throughput and valuable data meaning, it does not provide us with information about the spatial distribution of fluorescent signals or the way cells acquire the measured fluorescence. Therefore, the examined peptide might use various mechanisms to interact with cells. Peptides might cross the cell membrane to gain access inside cells, both via active or passive transport mechanisms, or they might associate with the cell surface or be inserted within the cell membrane. Further studies, which are beyond the scope of this work, are needed in order to ascertain more detailed information.

### 2.4. Effects of Peptides on HUVEC: Viability and Apoptosis Assays

It is noteworthy to observe that cell culture conditions such as medium composition, treatment concentrations and times, can strongly influence the cellular response. Based on this consideration, we tested different culture conditions and media containing different amounts of growth factor, to investigate the impact of the studied peptides on HUVEC cells. VEGFQ79G, VEGFI83G, VEGF73-101, and VEGF84-101 peptides were assayed for 24 h or 48 h in either survival (EBM2 basal medium, 0.2% FBS (Fetal Bovine Serum) without growth factors) or proliferation conditions (EBM2 basal medium, 2% FBS in the presence of growth factors, but in the absence of VEGF165) (see the Experimental Section for details).

**Figure 4:**
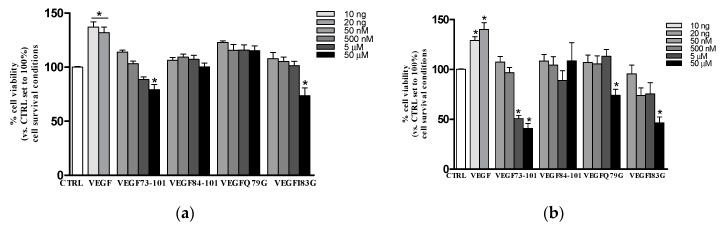
Effects of VEGF165 (VEGF 10-20 ng/mL), VEGF73-101, VEGF84-101, VEGFQ79G and VEGFI83G (peptides concentration range from 50 nM to 50 µM) on HUVEC viability. Cells (3× 10^3^ cells/wells) were treated for (**a**) 24 h and (**b**) 48 h in EMB2 basal medium with 0.2% FBS (Fetal Bovine Serum) without growth factors. At the end, the cell viability was evaluated by MTS (3-(4,5-dimethylthiazol-2-yl)-5-(3-carboxymethoxyphenyl)-2-(4-sulfophenyl)-2H-tetrazolium, inner salt) assay. Values are expressed as the percentage of viable cells with respect to untreated cells (CTRL). Data are the mean ± SEM of three different experiments performed in triplicate. Statistically significant differences are indicated with * = *p* < 0.05 vs. CTRL (One-Way ANOVA + Dunnett test).

In Figure 4, the dose-response curves in survival conditions are reported. The VEGF165, used as the positive control, significantly increased HUVEC viability at 24 h (about 20%, Figure 4a) and 48 h (about 40%, Figure 4b). Cell viability was not affected by peptides after 24 h of treatment. Conversely, at the higher dose tested (50 µM), all peptides (except VEGF84-101) were able to reduce cell viability, in particular VEGF73-101 and VEGF183G starting at 24 h and VEGFQ79G starting at 48 h. Notably, VEGF73-101 was the only one to significantly decrease cell viability also when used at 5 µM for 48 h, as reported in our previous paper [34].

In proliferation conditions, none of the investigated peptides produced a significant effect. The only one that showed the ability to increase cell viability at 24 h and 48 h was the full length VEGF165, as reported in the supplementary materials (Figure S3).

**Figure 5:**
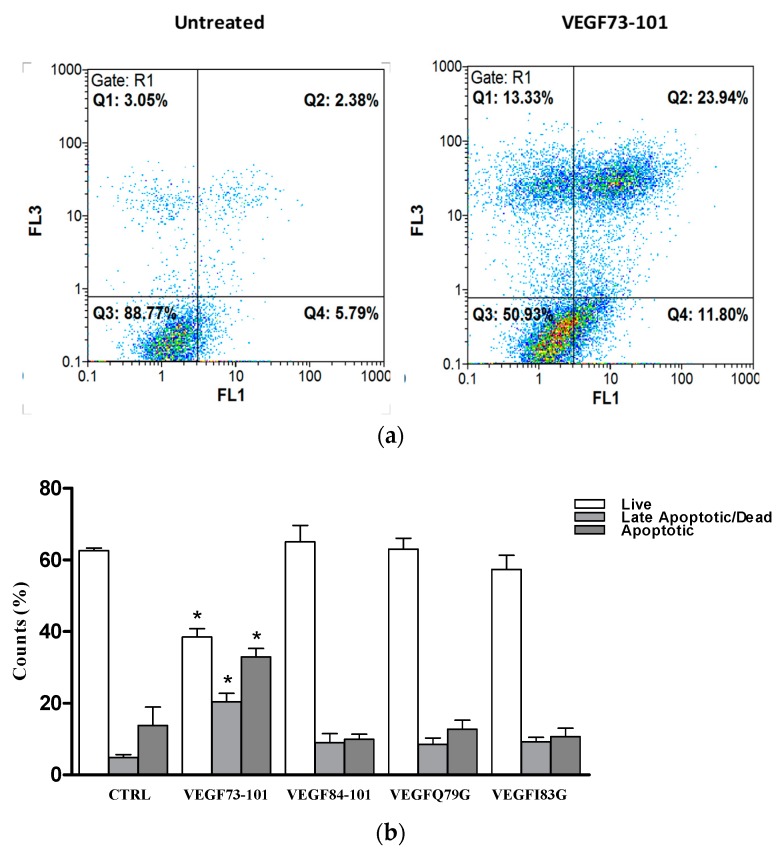
(**a**) Representative dot blot of HUVEC cells untreated (left) or exposed to VEGF73-101 (right) for 72 h; (**b**) the amount of viable, apoptotic and late apoptotic/dead HUVEC cells treated with VEGF73-101, VEGF84-101, VEGFQ79G, and VEGFI83G (5 µM), compared to untreated cells and those treated with STS (staurosporine) added 2 h before analysis. Data are the mean ± SEM of two different experiments performed in triplicate. Statistically significant differences are indicated with * = *p* < 0.05 vs. CTRL (One-Way ANOVA + Tukey’s test Student-Newman-Keuls test).

In order to provide some insight into the MTS (3-(4,5-dimethylthiazol-2-yl)-5-(3-carboxymethoxyphenyl)-2-(4-sulfophenyl)-2H-tetrazolium, inner salt) results, we performed PI/Annexin V assays. STS (staurosporine) was used as a positive control, being a well know apoptotic inducer [40]. The results reported in Figure 5 indicate, once more, that only VEGF73-101 was able to induce apoptosis, confirming the results obtained by the MTS assay in survival conditions. Neither the shorter sequence (VEGF84-101), nor the single mutated peptides induced apoptosis [34], indicating that the β-sheet conformation based on the wild-type sequence of VEGF73-101, as well as the lipophilicity of the VEGF73-101 sequence, are crucial to confer its pro-apoptotic features.

### 2.5. Effect of Copper (II) Ion on VEGFQ79G and VEGFI83G

As previously described, our study aimed to investigate the putative ionophoric activity of VEGF73-101, as well as establish a relationship between the structure of peptide fragments and the effect on cell viability in the presence of copper(II) ions. Therefore, we first compared the stoichiometry and conformational analysis of the VEGF73-101/Cu(II) complex with mutated peptides rich in histidine and sharing the copper binding region. These residues represented the main anchoring site of the metal in proteins and peptides.

To determine the metal binding stoichiometry, ESI-MS (Electrospray Mass Spectrometry) experiments were carried out. As shown in Table S1a,b, the spectra indicated the formation of multi-charged complexes of VEGFQ79G and VEGFI83G with copper(II) ions. Mono- and dinuclear copper complex species, together with their corresponding Na^+^ and K^+^ adducts, were observed. ESI spectra did not show qualitative changes upon the addition of one or more Cu(II) equivalents, both at acidic as well as at basic pH. As observed for VEGF73-101, the presence of dinuclear complex species was found at 0.8:1 metal to ligand molar ratio (Figure S4). By adding an excess of metal ion equivalents (up to three copper ion molar equivalents), traces of trinuclear copper (II) complexes were observed. However, in the presence of an excess of metal ions, precipitation partially took place by inducing an increase in signal noise, especially in the experiments performed at basic pH. Spectrometric results prevalently confirmed the presence of two major affinity sites for copper(II) ions. In Figure S4a,b, the relative percentages of the main species of VEGFQ79G/Cu and VEGFI83G/Cu complexes were reported. It is known that the abundance percentage of a species is affected by the capability of a compound to fly in the ESI source, so the differences found between the corresponding species of different peptides, VEGFQ79G and VEGFI83G, are only indicative and not absolute. However, for any compound, the ratio between different species in the same spectrum can be considered consistent. Bear in mind that the histidine residues were maintained in all peptides. VEGFQ79G and VEGFI83G showed a metal coordination mode similar to that of VEGF73-101 wild type peptide, in every experimental condition, as expected. To investigate the effect of metal ion binding on the peptide secondary structure far-UV CD spectra of VEGFQ79G/Cu(II) and VEGFI83G/Cu(II) complexes were carried out. The conformation of peptides in Figure S5 indicated a weak difference in signal intensity. In particular, the CD traces recorded with both 0.8:1 and 1.6:1 metal to ligand ratios showed a negative band centered at ~200 nm and a very small band centered at 225 nm, indicating a prevalent random-coil conformation with a band intensity unchanged with respect to that of the corresponding spectrum without metal (Figure 1).

The UV-Vis CD spectra of copper/peptide complexes in the visible region were used to gain insights from the metal ion d–d transitions about coordination geometry (Figure S6). The fragments VEGFQ79G and VEGFI83G, which had mutated sequences of VEGF73-101 to Q79 or I83 residues, respectively, preserved six protonation centers (Glu, 3His, and 2Lys), as well as the wild type peptide. Table 3, Table 4 and Table 5 show all spectroscopic results of copper complexes with the peptide at different pH values and L/M molar ratios.

**Figure 6:**
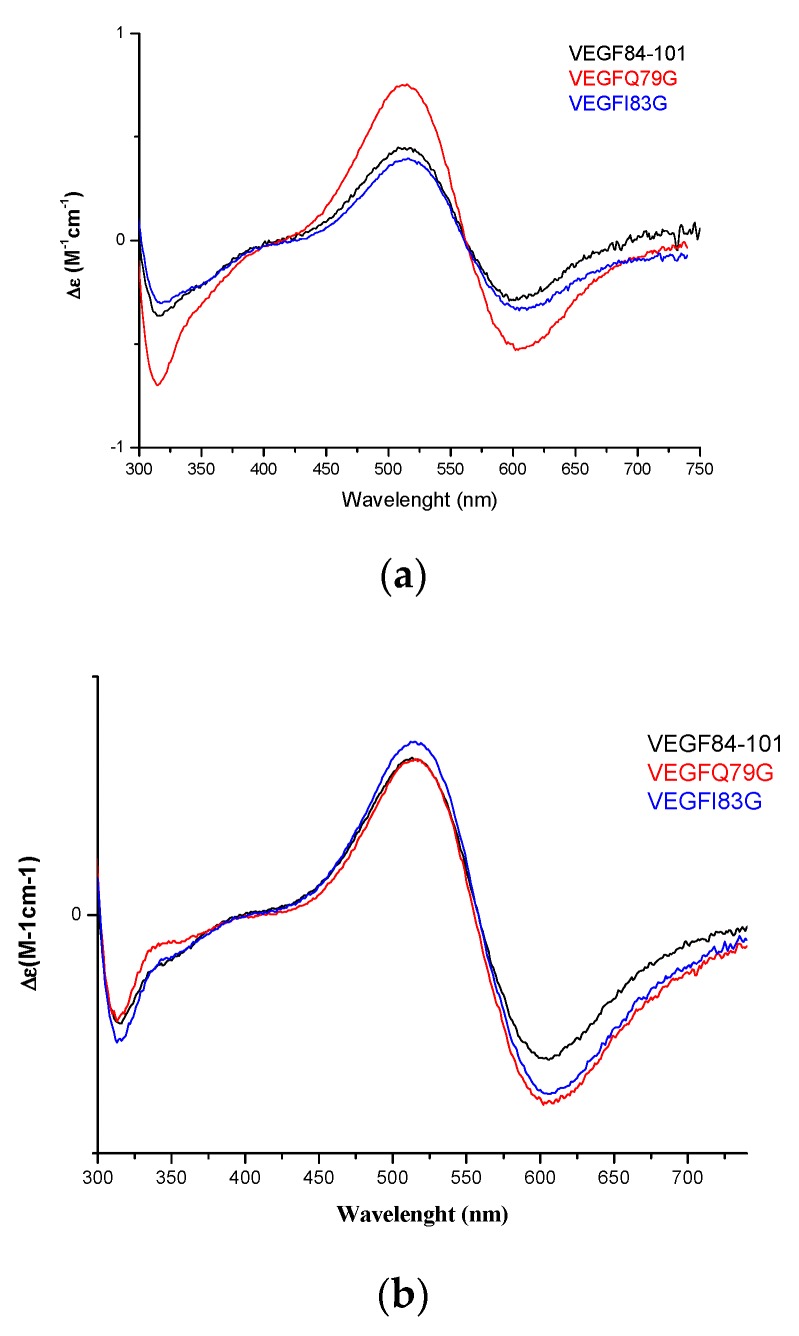

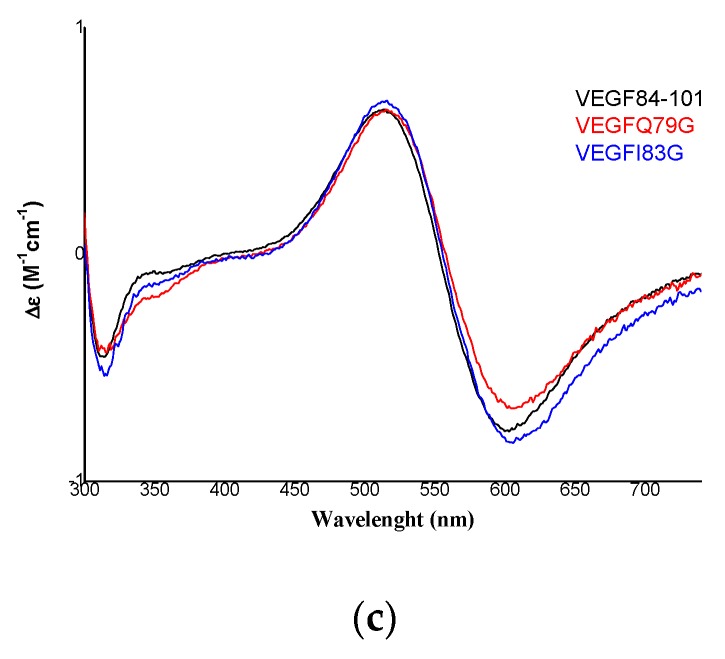
CD spectra of copper(II) complexes with VEGF84-101 [29], VEGFQ79G, VEGFI83G at pH 7 and 1:1 (**a**) 1.6:1 (**b**) 2.4:1 (**c**) metal to ligand molar ratio ([L] = 1 × 10^−3^ M).

The UV-Vis λ_max_ values of copper complexes showed a blue shift with increasing pH suggesting an increase in the number of donor atoms involved in the metal binding. When the metal to ligand ratio increased, changing from 0.8:1 to 2.4:1, the ε values significantly increased at pH 7. In agreement with the mass spectra findings, this indicated that two copper(II) ions bind to peptides. The UV-Vis CD spectra (Figure S7) showed two d–d transition bands centered around 605 nm and 514 nm only at pH 7, suggesting that the peptide backbone was affected at this pH value. The presence of a band with a minimum centered at 313 nm and a shoulder around 330 nm suggested the involvement of imidazole nitrogens and deprotonation amide coordinated to Cu(II). Figure 6 showed the UV-Vis CD spectra of the copper(II) complexes with VEGFQ79G, VEGFI83G and VEGF84-101, the latter already characterized by [34], at the same ligand/metal ratios and at physiological pH. Interestingly, all bands perfectly overlapped with each other, suggesting that mutated peptides have a very similar chiral environment and coordination mode to that of the VEGF84-101 and VEGF73-101, as reported in our previous work (3 N coordination mode with the contemporary involvement of deprotonated amide nitrogen and imidazole nitrogen more or less protonated: 2 N_Im_, N^-^_am_ and 1 N_Im_ protonated, or N _Im_, 2 N^-^ with the protonation of 2 N_Im_).

### 2.6. Model Membrane Assay of VEGF73-101/Cu(II) and Cellular Uptake

**Figure 7:**
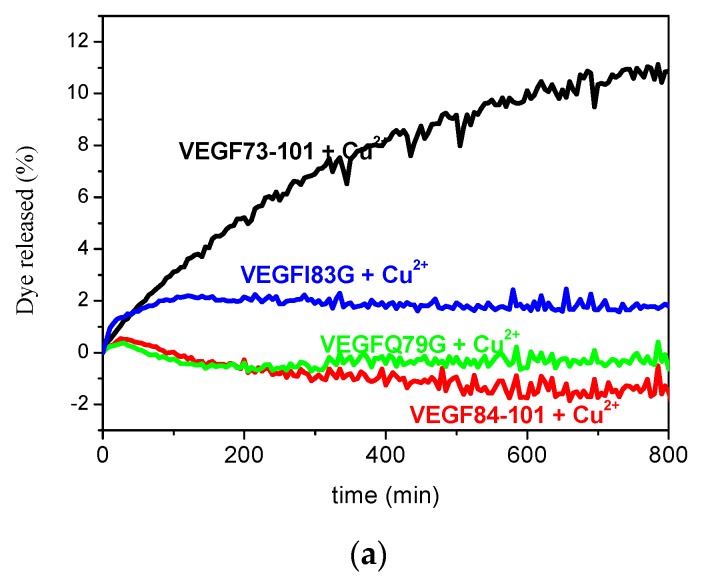

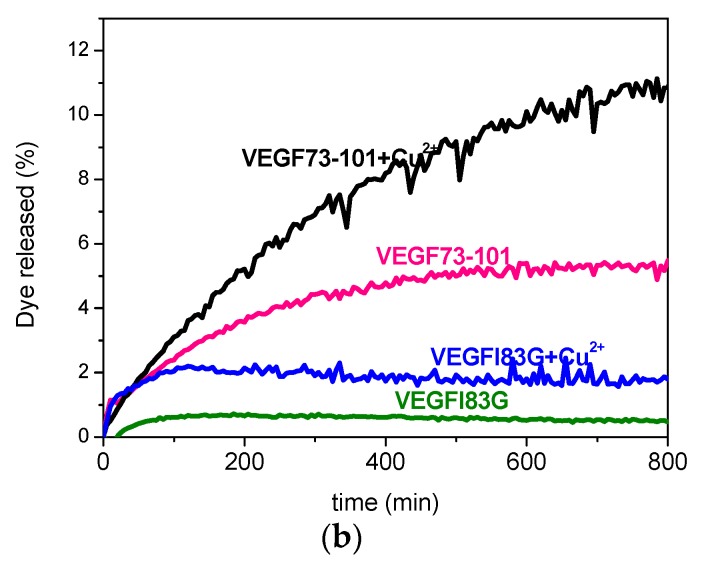
Peptide induced release of 6-carboxyfluorescein from 200 μM POPC:POPE:POPS LUVs measured in the presence of (**a**) 10 μM peptides/Cu and (**b**) 10 μM VEGF73-101 (magenta curve) and its 1:1 Cu(II) complex (black curve), 10 μM VEGFI83G (green curve) and its 1:1 Cu(II) complex (blue curve). Experiments were in triplicate at 25 °C in 10 mM phosphate buffer, 100 mM NaCl, pH 7.4.

The peptide–copper(II) complex membrane permeability was first investigated by monitoring the increase in fluorescent emission due to the release of 6-carboxyfluorescein from dye-encapsulated vesicles (Figure 7). We observed that only the 1:1 VEGF73-101/Cu(II) complex permeated model membranes more efficiently than the metal-free peptide (Figure 7), confirming a different behavior of wild type peptide with respect to the other peptides studied, although VEGFI83G copper complexes also weakly perturbed the membrane. Moreover, to investigate the conformational changes of membrane–peptide binding upon copper addition, Far-UV CD spectra were carried out. A membrane perturbation due to the metal contribution showed only for the VEGF73-101/Cu(II) complexes, which exhibited an increase in signal relative to β-sheet conformation with respect to the non-complexed peptide (Figure S8). The obtained results sustain the hypothesis that VEGF73-101 can act as an effective metal ionophore.

**Figure 8:**
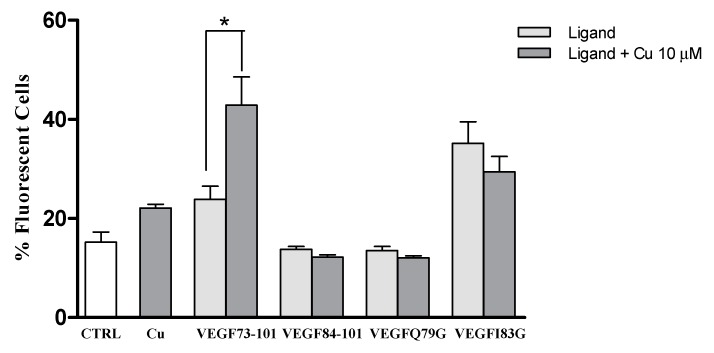
Histograms showing the number of fluorescent cells, expressed as %, of the whole population treated with the above indicated FAM-conjugated peptides (500 nM) and FAM-peptides/copper complexes. Data are the mean ± SEM of three different experiments performed in triplicate. Statistically significant differences are indicated with * = *p* < 0.05 vs. CTRL (One-Way ANOVA + Tukey’s test).

Many studies have demonstrated that in pathological conditions involving an angiogenetic process, cancerous cells have elevated copper concentrations [2,3,4]. Therefore, to verify whether or not copper also affected the ability of peptides to interact with membrane in living cells, we ran cytofluorimetric measurements on cells exposed to FAM-labeled (conjugated) peptides previously incubated with copper to produce peptide/Cu(II) complexes. To this end, VEGF73-101, VEGF84-101, VEGFQ79G and VEGFI83G peptides were premixed in water with copper sulfate (10 µM). Then the cells were exposed to peptides or peptide/Cu(II) complexes in the same conditions described in Section 2.3. To better evaluate the effect of metal complexation in these experimental conditions, the peptide concentration was aptly chosen at 500 nM (Figure 8), excluding the values that induced a reduction on cellular viability, 5 µM and 50 µM (Figure 4).

Figure 8 shows, that only cells exposed to the VEGF73-101/Cu(II) complexes, revealed an increase in the percentage of fluorescent cells, indicating that VEGF73-101/Cu(II) complexes interacted more with the membrane than their VEGF73-101 counterpart. None of the other peptides examined showed similar features.

### 2.7. Effects of Peptide/Copper(II) Complexes on HUVEC: Viability and Apoptosis Assays

**Figure 9:**
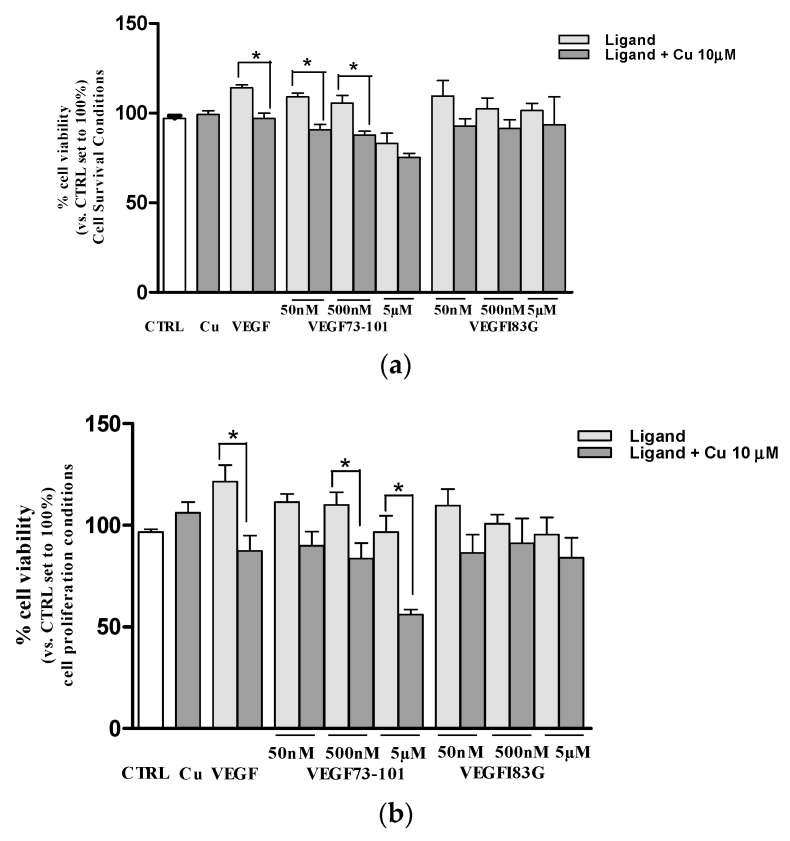
Evaluation of copper effects on VEGF165 (10 ng/mL), VEGF73-101, and VEGFI83G on HUVEC cells. (**a**) Cells (3 × 10^3^ cells/wells) were treated for 24 h in EMB2 basal medium with 0.2% FBS without growth factors. (**b**) Cells (1.5 × 10^3^ cells/wells) were treated for 24 h in EMB2 basal medium with 2% FBS with growth factors and without VEGF165. In the end, cell viability was evaluated by MTS assay. Values are expressed as the percentage of viable cells with respect to untreated cells (CTRL). Data are the mean ± SEM of three different experiments performed in triplicate. Statistically significant differences are indicated with * = *p* < 0.05 vs. CTRL (One-Way ANOVA + Tukey’s test).

Based on the results described in the Section 2.6, the presence of copper significantly influenced the interaction of VEGF73-101 and VEGFI83G with the cellular membrane in living cells, although VEGFI83G was less effective than VEGF73-101. For these reasons, the effects on cell viability of both the VEGF73-101/Cu(II) and VEGFI83G/Cu(II) complexes was examined by MTS assay, either in *survival* conditions or in *proliferation* conditions (Figure 9).

In both conditions examined (survival and proliferation), after 24 h, the VEGF73-101/Cu(II) complexes worsened the impact on cell viability with respect to the non-complexed counterpart, suggesting an increased toxicity caused by the formation of peptide/Cu(II) complexes. In the same experimental conditions, in fact, the corresponding peptide and the Cu(II) ion concentrations by themselves had no effects (Figure 9). Surprisingly, the presence of Cu(II) also reduced the percentage of live cells stimulated with VEGF165 protein (about 35% in proliferation conditions).

After 48 h of treatment (Figure S9), the VEGF73-101/Cu(II) complexes still decreased cell viability more than their non-complexed counterpart, although this result was less remarkable than at 24 h and occurred in proliferation conditions only. Both the VEGF165 protein and VEGF165/Cu(II) complexes did not show significant effects after 48 h, either in the survival or proliferation conditions examined by the MTS assay (Figure S9). To explain this behavior, we can speculate that for longer treatment times (more than 48 h), cells in the presence of VEGF165 seemed to adopt some compensative mechanisms (Figure S9) in order to counteract the toxicity induced by copper(II) supplementation. In all the experimental conditions described, the VEGFI83G/Cu(II) complexes did not produce any significant effect, demonstrating the relevance of the peptide structure.

**Figure 10:**
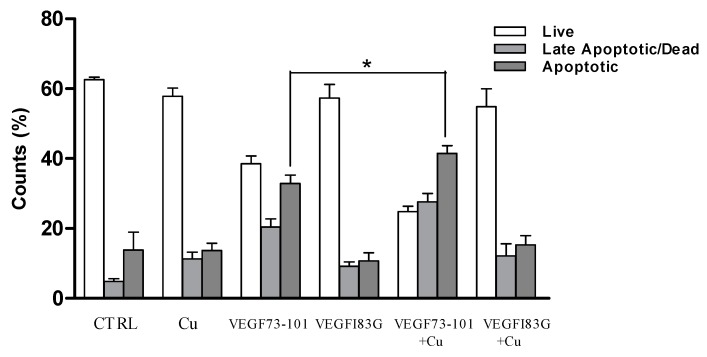
The viable, apoptotic and late apoptotic/dead populations of HUVEC cells previously treated with copper (II) 10 µM, peptides 5 µM, and peptide/Cu(II) complexes for 72 h, compared to untreated cells and those treated with STS (staurosporine) added 2 h before analysis. Data are the mean ± SEM of three different experiments performed in triplicate. Statistically significant differences are indicated with * = *p* < 0.05 VEGF73-101 vs. VEGF73-101 + Cu (One-Way ANOVA + Student-Newman-Keuls test).

Once again, the toxicity evidenced by the MTS assay for the peptide/copper complexes over the non-complexed counterparts was further investigated using the PI/Annexin V assay (Figure 10). In fact, only the VEGF73-101 peptide in the presence of Cu(II) caused a decrease in live cells, supporting the importance of the lipophilicity and β-sheet conformation of the VEGF fragment structure. Notably, the increase in copper levels was related to an increase in oxidative stress and apoptosis in different cell types [41,42,43]. Therefore, the presence of VEGF73-101/Cu(II) complexes might result in increased levels of intracellular copper, accounting for the raised apoptotic rate observed. However, more experiments are needed to better decipher these results.

### 2.8. Effect of a Copper Chelator (BCS) on Cells HUVEC Treated with VEGF73-101/Cu(II)

To verify if the copper amount in the medium could influence peptide activity, we performed MTS assay pretreating cells with a known membrane-impermeable copper chelator, bathocuproine disulfonic acid disodium salt (BCS).

According to [44], the response induced by copper ions in endothelial cells is strongly determined by the culture condition settings. Copper salts are an essential component of culture media with concentration ranges from 0.94 µM to 1.9 µM [44]. Although they come in small quantities, this basal amount is not negligible, as confirmed by [45,46]. As shown in Figure S10, VEGF73-101’s effect on cell viability was not significantly altered by BCS, suggesting that the amount of copper in the EBM-2 basal medium was not able to significantly alter peptide activity (Figure S10).

**Figure 11:**
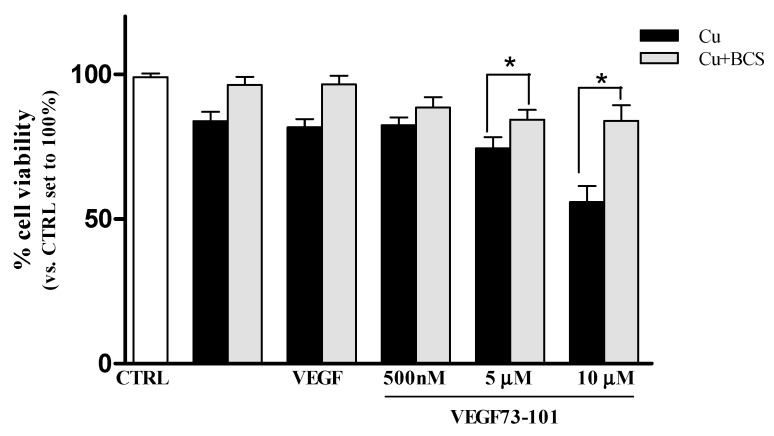
Effects of copper chelation on VEGF165 (10 ng/mL) and VEGF73-101 on HUVEC. Cells (1.5 × 10^3^ cells/wells) were treated for 24 h in EMB2 basal medium with 2% FBS with growth factors without VEGF165, in the presence of 10 µM Cu(II), without or with bathocuproine disulfonic acid disodium salt (BCS) (50 µM). In the end, cell viability was evaluated by MTS assay. Values are expressed as the percentage of cell viability in accordance with their respective untreated cells (CTRL). Data are the mean ± SEM of three different experiments performed in triplicate. Statistically significant differences are indicated with * = *p* < 0.05 VEGF73-101-Cu vs. VEGF73-101-Cu/BCS (One-Way ANOVA + Tukey’s test).

To highlight the importance of copper complexation with the peptide chain on HUVEC cell viability, we performed MTS assay pretreating the cells with BCS at 50 μM for 24 h. As can be observed in Figure 11, the decrease in viability of cell samples treated with different doses of peptide/Cu(II) complexes was counteracted by the pretreatment with BCS. These results seem to indicate that the VEGF73-101 peptide can behave as a “metal ionophore” [47,48,49,50,51].

## 3. Materials and Methods

### 3.1. Materials

Human umbilical vein endothelial cells (HUVECs) and cell culture media (EGM^®^-2 BulletKit^®^, culture system containing EBM-2 (Endothelial Cell Growth Basal Medium) and EGM-2 SingleQuots supplements required for growth of endothelial cells) were purchased from Lonza srl (Milan, Italy). Recombinant Human VEGF165 was obtained from PeproTech (5 Crescent Ave, Rocky Hill, NJ, USA). Bathocuproine disulfonic acid disodium salt and 6-carboxyfluorescein were purchased from Fluka, Sigma-Aldrich (Milan (MI), Italy). VEGF73-101 and VEGF84-101 were synthesized as reported in previous work [32], while VEGFQ79G and VEGFI83G were purchased from Genecust (Dudelange, Luxembourg). N- and C-terminally blocked fragments. 1,2-palmitoyl-oleoyl-sn-glycero-3-phosphocholine (POPC), 1,2-palmitoyl-oleoyl-sn-glycero-3-phosphocholine (POPE) and 1,2-palmitoyl-oleoyl-sn-glycero-3-phospho-L-serine (POPS) were purchased from Avanti Polar Lipids (Alabaster Alabama (AL), USA) with a purity of 99% and used without further purification.

### 3.2. Spectroscopic Measurements (CD e UV)

The UV-Vis spectra of copper(II) complexes were recorded at 25 °C using a UV-Vis/NIR Jasco V-670 spectrophotometer using 1 cm path length quartz cells, in the same concentration range used for CD measurements, using a 1.0 mM peptide concentration at different metal to ligand ratios, 0.8:1, 1.6:1 and 2.4:1. Maximum wavelength (λ) and molar coefficient (ε) values refer to a single species when this is the only one that forms in the experiment used.

CD spectra were obtained at 25 °C under a constant flow of nitrogen on a Jasco model 810 spectropolarimeter at a scan rate of 50 nm min^−1^ and a resolution of 0.1 nm. The path length was 1 cm. All the solutions were freshly prepared using double distilled water. The CD spectra of peptides and copper(II) complexes, upon varying the solution pH, were obtained in the range 190–350 at 5 µM and in the range 240–750 nm at 1 mM by employing different metal to ligand ratios (0.8:1, 1.6:1, and 2.4:1). The spectra were recorded as an average of 10. Calibration of the instrument was performed with a 0.06% solution of ammonium camphorsulfonate in water.

### 3.3. Cell Cultures

HUVECs were maintained in EGM^®^-2 BulletKit^®^ medium. Cells were grown to 80% confluence at 37 °C in a humid atmosphere in the presence of 5% CO_2_. Passages 2–5 were used. Cells were starved for 12 h, using endothelial cell basal medium (EBM^®^-2 basal medium, 0,2% Fetal Bovine Serum) before all experiments.

### 3.4. Cell Viability Assay (MTS)

The MTS assay (CellTiter 96^®^ AQueous One Solution, Promega) was performed to compare the viability of cells treated with different concentrations of peptides (VEGF73-101, VEGF84-101 VEGFQ79G, and VEGFI83G) and CuSO_4_, and their complexes. We considered two different growth conditions for our treatments to be examined, to which we refer in this paper as proliferation conditions or survival conditions. Proliferation condition: Cells (3 replicates) were seeded in 96-well plates at a density of 1.5 × 10^3^ cell/well, treated in EGM^®^-2 BulletKit^®^, 2% Fetal Bovine Serum (FBS) without VEGF supplementation. Survival condition: Cells (3 replicates) were seeded in 96-well plates at a density of 3 × 10^3^ cell/well in an EBM^®^-2 basal medium of 0.2% FBS without supplements and VEGF. MTS assay was used following 24 h or 48 h of treatments according to the manufacturer’s instructions. The required copper and peptides were pre-mixed together in water in order to allow peptide/copper complexes formations before their addition to the cells. When the experiments were performed in the presence of bathocuproine disulfonic acid disodium salt (BCS), in proliferation condition, 50 µM of the copper chelator was added during the treatment for 24 h. The results were reported as the percentage of viable cells, with respect to the control set, to 100%.

### 3.5. Annexin V-FITC/PI Staining

Annexin V/ PI is a commonly used approach for studying apoptotic cells. Propidium iodide (PI) is widely used in conjunction with Annexin V to determine if cells are viable, apoptotic, or necrotic through differences in plasma membrane integrity and permeability. The fluorescence of AnnexinV-FITC/PI assay was read using flow cytometry. HUVEC cells were seeded on a 12-well plate at a density of 2.5 ×10^4^ and grown for 48 h before treatments. During the last 24 h cells were starved in the same EBM-2 basal medium containing 0.2% fetal bovine serum without EC (endothelial cell) supplements. Cells were then exposed to 5 µM peptide for 72 h. When required, 10 µM CuSO_4_ was used in combination with peptides. Five hours of incubation in the presence of 1 µM STS (staurosporine) was used as a positive control for cell death. Cells were harvested and stained with Annexin V-FITC (Fluorescein isothiocyanate) and propidium iodide using an annexin V-FITC apoptosis detection kit (Life technologies, Monza (MB), Italy), following the manufacturer’s instructions. Briefly, cells were pelleted and resuspended in 800 µL PBS buffer containing 20 µL of annexin V, 20 µL of propidium iodide, and PI (Annexin V/Dead Cell Apoptosis Kit (Invitrogen), and kept for 10 min at 25 °C in the dark. Samples were analyzed within 1 h by flow cytometry using a CyFlow^®^ ML (Sysmex Partec Milan-Italy). The fluorescent scatter plots indicated three types of cell populations: viable (Annexin V-FITC−/PI−), dead (Annexin V-FITC + /PI+), and apoptotic (Annexin V-FITC + /PI−). Quadrant analysis (FlowMax software -Partec) was performed on the gated fluorescent scatter plot to examine the percentage of live, dead, and apoptotic cell populations. Experiments were repeated twice in triplicate.

### 3.6. Flow Cytometry

Sample were analyzed, 2 × 10^4^ cells per sample, using a CyFlow^®^ ML flow cytometer (Partec) system equipped with three laser sources and 10 optical parameters with dedicated filter setting and a high numerical aperture microscope objective (50× NA 0.82) for the detection of different scatter and fluorescence signals. Sample gating, based on forward and side scatter of 2 × 10^4^ cells, was included for analysis. Cells were excited by an air-cooled argon 488 nm laser before the signal from FAM or FITC was read on the FL1 detector and the signal from PI on the FL3 detector. Data obtained were acquired, gated, compensated, and analyzed using the FlowMax software (Sysmex Partec, Milan, Italy).

### 3.7. Interaction Peptides/Cell Membranes in Living Cells

HUVEC cells, 4.5 × 10^4^, were seeded on a 6-well plate. Cells were treated with FAM-conjugated peptides for 24 h, 24 h after seeding. Cells were harvested by trypsinization and resuspended in Krebs ringer buffered saline (130 mM NaCl, 3.6 mM KCl, 10 mM HEPES, 2 mM NaHCO_3_, 0.5 mM NaH_2_PO_4_, 0.5 mM MgCl_2_, 1.5 mM CaCl_2_, 4.5 g/L glucose, pH 7.4). Cells were then analyzed by flow cytometry on FL1 log mode. Data reported are representative of three sets of independent experiments, each performed in triplicate and based on 20,000 events for each group.

### 3.8. Model Membrane Permeabilization Assays

Model membranes were prepared according to a procedure described elsewhere [52]. To mimic the lipid composition of endothelial cell membranes, we used POPC:POPE:POPS (5:3:2 molar ratio) lipid mixture [53]. Multilamellar vesicles (MLVs) were obtained by vortexing a chloroform lipid solution under a gentle nitrogen flow. Next, the lipid solution was dried overnight in a vacuum at room temperature in a round-bottomed flask to remove all the residual solvent. The resulting lipid film was hydrated with a proper amount of 10 mM phosphate buffer, pH 7.4, NaCl 100 mM and dispersed by vigorous stirring. Large unilamellar vesicles (LUVs), were obtained by extruding the MLVs through polycarbonate filters (pore size = 100 nm, Nuclepore, Pleasanton, CA, USA) mounted in a mini-extruder (Avestin, Ottawa, ON, Canada) fitted with two 0.5 mL Hamilton gastight syringes (Hamilton, Reno, NV, USA). Peptide-induced membrane permeabilization was investigated using large unilamellar vesicles (LUVs) filled with a fluorescent dye (6-carboxyfluorescein). Pore formation in the lipid bilayer was monitored by measuring the increase in fluorescence emission intensity due to dye dilution (dequenching) in the buffer upon membrane leakage. Dye-filled POPC:POPE:POPS LUVs were prepared by hydrating the dry lipid film with a 10 mM phosphate buffer solution containing 70 mM 6-carboxyfluorescein. The non-encapsulated dye was removed by placing the solution containing LUVs on a Sephadex G50 gel permeation column (Sigma-Aldrich, Milan (MI), Italy) and eluting it by final buffer (10 mM phosphate, 100 mM NaCl, pH 7.4) in order to ensure the balance of the osmotic pressure in the lipid bilayer. The first colored band, including the separated dye-containing vesicles, was collected. The final lipid concentration (200 µM) was checked using the Stewart assay [54]. Dye-leakage experiments were carried out in 96-well plates. Samples were prepared by adding the corresponding amount of peptide/Cu^2+^ solution (peptide final concentration 10 µM; peptide/Cu molar ratio 1:1 or 1:2) to 100 µL of LUV (POPC:POPE:POPS (5:3:2), 200 µM in buffer phosphate 10mM, pH 7.4, NaCl 100 mM. Time traces were recorded using a Varioskan plate reader (ThermoFisher, Waltham, MA, USA) with λ_ex_ = 494 nm and λ_em_ = 520 nm at 25 °C. The fraction of dye release (or leaked membrane) was calculated according to the following Equation (1):

(1) $$Percentage\;of\;leaked\;membrane=\frac{\left(I-I_{0}\right)}{\left(I_{100}-I_{0}\right)}$$

where *I* is the fluorescence emission of the sample, *I*_0_ is the emission intensity obtained in the absence of peptide (baseline control), and *I*_100_ is the emission intensity obtained after addition of the detergent Triton X-100, which caused 100% leakage. All measurements were the average of three replicated experiments.

### 3.9. Electrospray Mass Spectrometry (ESI-MS) Analysis

The high-resolution mass spectrometer used was an Orbitrap Q-Exactive (Thermo Scientific, Milan, Italy) equipped with an HESI II interface set in positive (ESI(+)) polarity. A Q-Exactive mass spectrometer was operated in full scan (70 000 FWHM). Mass spectra were recorded from m/z 400 to 2000. Acquisition parameters were optimized by direct infusion of standard working solutions of the analytes (50 µM in water) at a flow rate of 3 μL min^−1^ into the ESI interface of the mass spectrometer. The parameters were optimized as follows: spray voltage set at 3.5 kV (ESI(+)), capillary temperature set at 300 °C; sheath, auxiliary, and sweep gases (nitrogen) fixed at 5, 0, and 0 (arbitrary unit), respectively. Acquisition data were recorded and elaborated using Xcalibur™ version 3.0 software from Thermo Fisher.

### 3.10. UHPLC-ESI MS

To evaluate the relative hydrophobicity of the peptides, the retention times of amidated-acetylated peptides were recorded on a PepMap C18 column 300Å (75 μm × 150 mm) using a linear gradient from 2% to 99% of B (20% water–80% acetonitrile) containing 0.1% FA over 40 min, at a flow rate of 0.3 µL/min. The injection volume was 5 μL for analysis. The Q-exactive mass spectrometer was equipped with nano electrospray ion source (Nano EASY-Spray). The thermostatted column compartment was at 40 °C. The optimized parameters of mass spectrometry were illustrated as below: spray voltage, + 3.0 kV; capillary temperature, 250 °C; full MS; resolution, 70,000; automatic gain control (AGC) target, 3.0 × 10^6^; maximum injection time (IT), 200 ms; scan range, 150–2000m/z. Nitrogen was used for spray stabilization. All data collected in profile mode were acquired and processed using Thermo Xcalibur 3.0 software.

### 3.11. Statistical Analysis

Graph-Pad Prism (Version 5.00) was used for data analysis and graphic presentation. Data were reported as the mean ± SEM of at least three or two different experiments. Statistical analyses were performed using a one-way ANOVA study followed by the Tukey’s or Student-Newman-Keuls, and Dunnett test for repeated measurements. Differences were considered statistically significant when *p* < 0.05.

## 4. Conclusions

Copper is indispensable in the angiogenesis process, the administration of copper sequestering agents as an antitumor approach is under evaluation [5,6,7]. During the angiogenesis process, copper translocates from intracellular to extracellular space [55]. The portion of copper that remains complexed is transferred following diffusion law [18,19,21] or by giving the metal to transporters or chaperones [20,56]. In the cells there are high concentrations of both proteins and ligands that have high-affinity Cu(II)-binding, but that cannot pass the plasma membrane freely. Conversely, some compounds are capable of complexing the metal and freely traversing the various compartments, according to different kinetics conditioning their effectiveness. Metal ionophores may induce cellular toxicity with a great number of mechanisms, and understanding could prove to be advantageous for transforming the most interesting compounds into clinically useful therapeutics agents [57].

In this study, the ionophoretic activity of the pro-apoptotic bifunctional peptide, which can both chelate the Cu(II) ions and inhibit HUVEC viability, was evaluated.

For this purpose, two peptides singly mutated and aptly designed, were characterized. The new peptides showed different characteristics with respect to the native peptide, both for chemical–physical properties and for the biological activities. In particular, the loss of glutamine residue in the mutated Q79G, key in the interaction with VEGR2, induced a perturbation of the charges and spatial conformation. However, both fragments confirmed that the punctual variation of an amino acid determined the change of the native peptide conformation, shifting from predominantly β-sheet to predominantly random coil. Additionally, the single amino acid mutation was able to perturb the lipophilic properties of the peptide. Therefore, VEGF73-101 biological activity seemed to depend on the set of several factors, including its different conformation as well as its hydrophobicity. Decidedly, the assays on artificial membranes confirmed that only the VEGF73-101 exhibited a strong perturbation of the model membrane, and suggested that the peptide could cause damage to the cellular membrane.

Interestingly, our results showed a correlation between the lipophilic character of the ligands and an increase in the cytotoxic activity of the corresponding copper complexes. The region, including the hydrophobic residues, resulted in a determining factor for peptides/membrane interactions.

Moreover, the supplementation of copper amplified the artificial membrane damage VEGF73-101 induced, and intensified the cell viability reduction and the pro-apoptotic effect. Furthermore, the BCS chelating agent counteracted the copper-induced toxic effect, supporting the idea that the effects of this peptide on HUVEC cell viability were also due to alterations in copper ion availability. This recovery suggests BCS’s ability to subtract metal from VEGF73-101/copper complexes and the ability of the peptide to chelate Cu(II) on cells.

In conclusion, we showed that VEGF73-101 behaves as a copper ionophore and represents an interesting candidate in the discovery and development of new peptides in the designing of effective anti-angiogenic therapies.

## Figures and Tables

**Table 1:** Amino acid sequences of VEGFfragments and mutated peptides.

Peptide Sequences	
Ac-ESNITM**Q**IMR**I**KPHQGQHIGEMSFLQHNK-NH2	**VEGF73-101**
Ac-ESTNIM**G**IMR**I**KPHQGQHIGEMSFLQHNK-NH2	**VEGFQ79G**
Ac-ESTNIM**Q**IMR**G**KPHQGQHIGEMSFLQHNK-NH2	**VEGFI83G**
Ac-KPHQGQHIGEMSFLQHNK-NH2	**VEGF84-101**

Residues involved in VEGF/VEGFR2 binding are shown in bold, point mutation is shown in underlined bold and histidine residues in red.

**Table 2:** Comparison of RP-HPLC retention time (t_R_) data of VEGF fragments and mutated peptides on PepMap C18 column by UHPLC-ESI MS.

Peptides	t_R_ (min)
VEGF73-101	21.7
VEGF84-101	18.2
VEGFQ79G	21.4
VEGFI83G	20.4

**Table 3:** Spectroscopic parameters of copper(II) complexes with VEGFQ79G and VEGFI83G. [L] = 1 × 10^−3^ M, 0.8:1 metal to ligand molar ratio at pH 5, 7, and 11.

pH	Ligand	UV/Vis λmax [nm] ε [M^−1^ cm^−1^])	CD λmax [nm] (Δε[Μ^−^^1^ cm^−1^])
7	VEGFQ79G	581(134)	605(−0.52) ^a^; 514(0.75) ^a^; 313(−0.68) ^b^
7	VEGFI83G	600(86)	605(−0.34) ^a^; 514(0.39) ^a^; 313(−0.28) ^b^

^a^ d–d Transition. ^b^ N_im_ →Cu^2+^ charge transfer.

**Table 4:** Spectroscopic parameters of copper(II) complexes with VEGFQ79G and VEGFI83G. [L] = 1 × 10^−3^ M, 1.6:1 metal to ligand molar ratio at pH 5, 7, and 11.

pH	Ligand	UV/Vis λmax [nm] (ε [M^−1^ cm^−1^])	CD λmax [nm] (Δε[Μ^−^^1^ cm^−1^])
7	VEGFQ79G	589(207)	605(−0.78) ^a^; 514(0.65) ^a^; 314(−0.44) ^b^
7	VEGFI83G	600(166)	605(−0.75) ^a^; 514(0.72) ^a^; 314(−0.52) ^b^

^a^ d–d Transition. ^b^ N_im_ →Cu^2+^ charge transfer.

**Table 5:** Spectroscopic parameters of copper(II) complexes with VEGFQ79G and VEGFI83G. [L] = 1 × 10^−3^ M, 2.4:1 metal to ligand molar ratio at pH 5, 7, and 11.

pH	Ligand	UV/Vis λmax [nm] (ε [M^−1^ cm^−1^])	CD λmax [nm] (Δε[M^−1^ cm^−1^])
7	VEGFQ79G	592(244)	606(−0.68) ^a^; 515(0.64) ^a^; 313(−0.41) ^b^
7	VEGFI83G	-----	606(−0.83) ^a^; 515(0.68) ^a^; 314(−0.53) ^b^

^a^ d–d Transition. ^b^ N_im_ →Cu^2+^ charge transfer.

## Supplementary Materials

Supplementary materials can be found at https://www.mdpi.com/1422-0067/21/8/2866/s1.
