# Supplementary material for: The Ionophoric Activity of a Pro-Apoptotic VEGF165 Fragment on HUVEC Cells
Source: Int J Mol Sci. 2020 Apr 20;21(8):2866. doi: 10.3390/ijms21082866 (PMC7216235; doi:10.3390/ijms21082866)
Supplement: Supplementary file 1 [file ijms-21-02866-s001.docx]

**Figure S1.** Representative UHPLC ESI-MS (TIC) chromatograms of peptides.

**Figure S2.** Far-UV CD comparison of peptides in water and in membrane.

|  |  |
| --- | --- |
| (**a**)   |  (**b**) |

**Figure S3.** Effects of VEGF165 (VEGF 10–20 ng/mL), VEGF73-101, VEGF84-101, VEGFQ79G and VEGFI83G (peptides concentration range from 50 nM to 50 µM) on HUVEC cell viability. Cells (1.5 × 10^3^ cells/wells) were treated for (**a**) 24 h and (**b**) 48 h in EMB2 basal medium with 2% FBS with growth factors and without VEGF165. At the end, the cell viability was evaluated by MTS assay. Values are expressed as the percentage of viable cells in accordance with their untreated cells (CTRL). Data are the mean±SEM of three different experiments performed in triplicate. Statistically significant differences are indicated with asterisks:*=p<0.05 vs. CTRL (One-Way ANOVA + Dunnett test).

**Table 1. a.** Singly and multiple charged ions detected in the ESI mass spectra of VEGFQ79G and its mixtures with CuSO_4_ (0.8:1, 1.6:1 and 2.4:1 metal to ligand ratios).

| **Species** | **m/z _obs_** | **m/z _calc_** |
| --- | --- | --- |
| [L+2H]^2+^ | 1701.85 | 1701.84 |
| [L+Cu]^2+^ | 1732.31 | 1732.34 |
| [L+Cu+Na-H]^2+^ | 1743.30 | 1743.34 |
| [L+3H]^3+^ | 1134.91 | 1134.90 |
| [L+Na+2H]^3+^ | 1142.24 | 1142.23 |
| [L+Cu+H]^3+^ | 1155.22 | 1155.23 |
| [L+Cu+Na]^3+^ | 1162.54 | 1162.56 |
| [L+2Cu-H]^3+^ | 1175.19 | 1175.56 |
| [L+4H]^4+^ | 851.43 | 851.42 |
| [L+Na+3H]^4+^ | 856.93 | 856.92 |
| [L+2Na+2H]^4+^ | 862.43 | 862.42 |
| [L+Cu+2H]^4+^ | 866.43 | 866.67 |
| [L+Cu+Na+H]^4+^ | 872.16 | 872.17 |
| [L+Cu+2Na]^4+^ | 877.66 | 877.67 |
| [L+Cu+3Na-H]^4+^ | 883.15 | 883.17 |
| [L+2Cu]^4+^ | 881.89 | 881.92 |
| [L+2Cu+2Na-2H]^4+^ | 892.88 | 892.92 |
| [L+5H]^5+^ | 681.35 | 681.34 |
| [L+Cu+3H]^5+^ | 693.54 | 693.53 |
| [L+2Cu+H]^5+^ | 705.71 | 705.73 |
| [L+6H]^6+^ | 567.79 | 567.95 |
| [L+Cu+4H]^6+^ | 578.10 | 578.11 |
| [L+2Cu+2H]^6+^ | 588.26 | 588.28 |
| [L+3Cu]^6+^ | 598.41 | 598.44 |
| [L+Cu+5H]^7+^ | 495.67 | 495.67 |

**Table 1. Sb.** Singly and multiple charged ions detected in the ESI mass spectra of VEGFI83G and its mixtures with CuSO_4_ (0.8:1, 1.6:1 and 2.4:1 metal to ligand ratios).

| **Species** | **m/z _obs_** | **m/z _calc_** |
| --- | --- | --- |
| [L+2H]^2+^ | 1709.344 | 1709.332 |
| [L+Cu]^2+^ | 1739.794 | 1739.832 |
| [L+3H]^3+^ | 1139.897 | 1139.888 |
| [L+2H+Na]^3+^ | 1147.223 | 1147.221 |
| [L+Cu+H]^3+^ | 1160.202 | 1160.221 |
| [L+Cu+Na]^3+^ | 1167.529 | 1167.555 |
| [L+2Cu-H]^3+^ | 1180.510 | 1180.555 |
| [L+3Cu-3H]^3+^ | 1200.817 | 1200.888 |
| [L+4H]^4+^ | 855.173 | 855.166 |
| [L+Na+3H]^4+^ | 860.671 | 860.666 |
| [L+2Na+2H]^4+^ | 866.168 | 866.166 |
| [L+Cu+2H]^4+^ | 870.159 | 870.159 |
| [L+Cu+Na+H]^4+^ | 875.900 | 875.916 |
| [L+Cu+2Na]^4+^ | 881.397 | 881.416 |
| [L+Cu+3Na-H]^4+^ | 886.890 | 886.916 |
| [L+2Cu]^4+^ | 885.680 | 885.666 |
| [L+2Cu+2Na-2H]^4+^ | 896.623 | 896.666 |
| [L+5H]^5+^ | 684.340 | 684.333 |
| [L+Cu+3H]^5+^ | 696.523 | 696.533 |
| [L+2Cu+H]^5+^ | 708.705 | 708.733 |
| [L+6H]^6+^ | 570.452 | 570.450 |
| [L+Cu+4H]^6+^ | 580.603 | 580.610 |
| [L+2Cu+2H]^6+^ | 590.755 | 590.777 |
| [L+3Cu]^6+^ | 600.944 | 600.911 |
| [L+Cu+5H]^7+^ | 497.804 | 497.809 |

|  |
| --- |
| (**a**) |
| 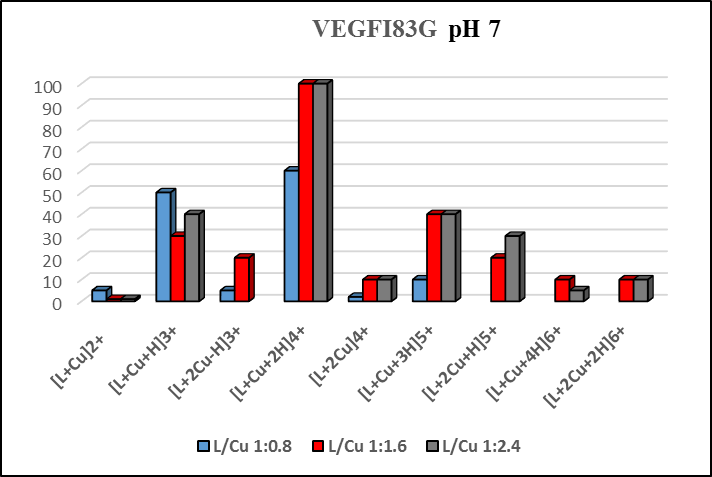 |
| (**b**) |

**Figure S4.** Relative abundance comparison of same species of (**a**) VEGFQ79G and (**b**) VEGFI83G peptide at different ligand to metal molar ratios (pH7).

| **** | **** |
| --- | --- |
| (**a**) | (**b**) |

**Figure S5.** UV-CD spectra of copper (II) complexes with (**a**)VEGFQ79G and (**b**)VEGFI83G at 1.6:1 metal to ligand molar ratio ([L]= 1× 10^−5^ M), 5-10 pH range.

| 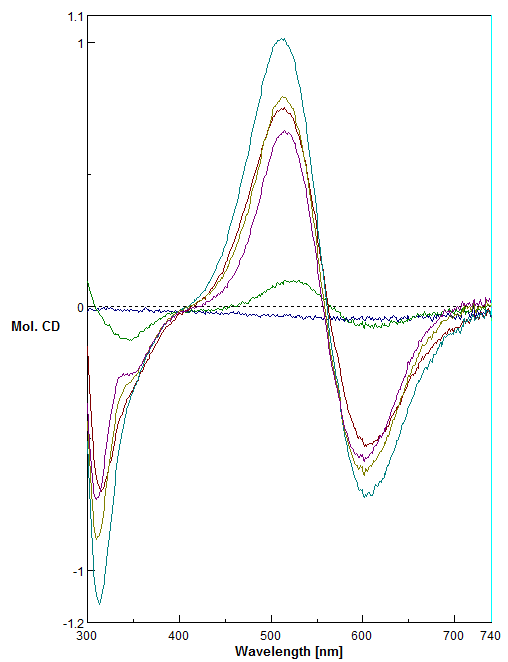 | 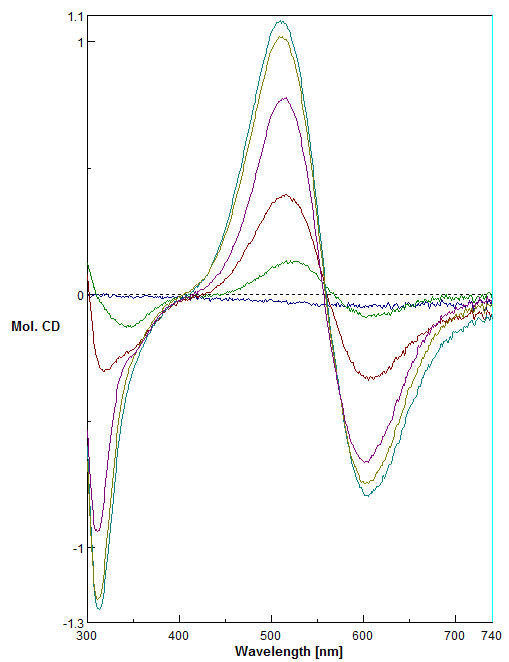 |
| --- | --- |
| (**a**) | (**b**) |
| 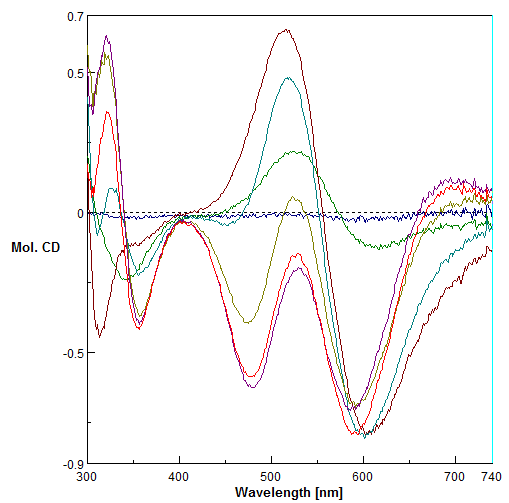 | 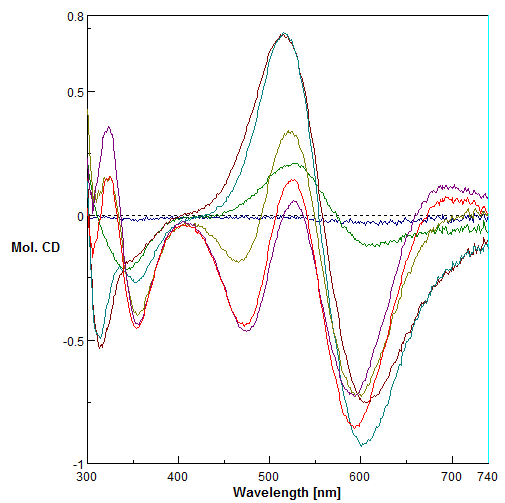 |
| (**c**) | (**d**) |
| 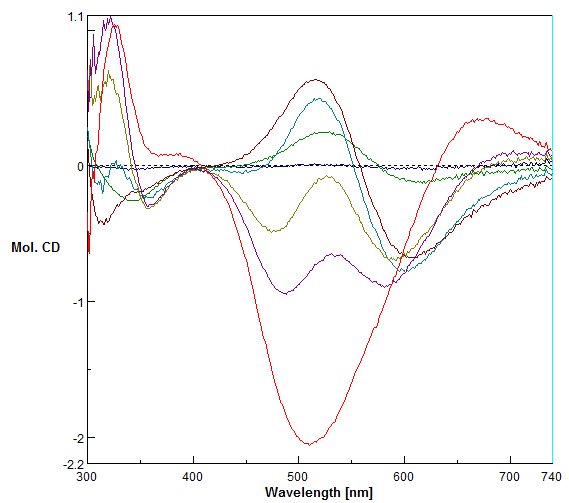 | 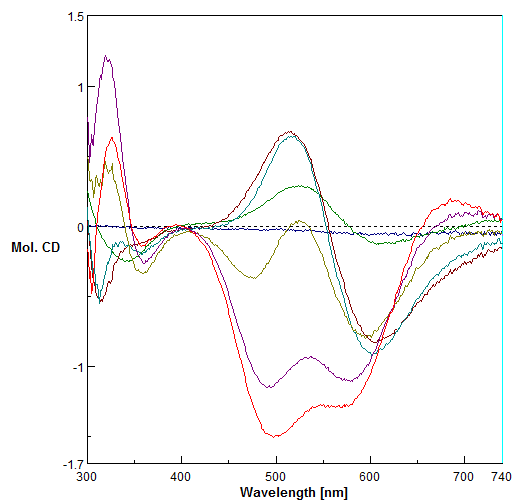 |
| (**e**) | (**f**) |

**Figure S6.** CD spectra of copper (II) complexes with VEGFQ79G at (**a**)0.8:1, (**c**)1.6:1, (**e**)2.4:1 and VEGFI83G at (**b**)0.8:1, (**d**)1.6:1, (**f**)2.4:1 metal to ligand molar ratio ([L]= 1 × 10^−3^ M), 5<pH<11.

VEGFQ79G+Cu 0.8:1

VEGFQ79G +Cu 1.6:1


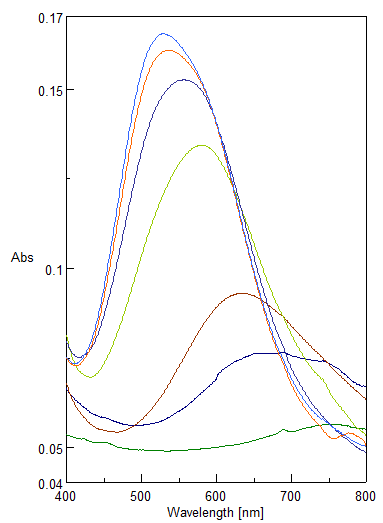

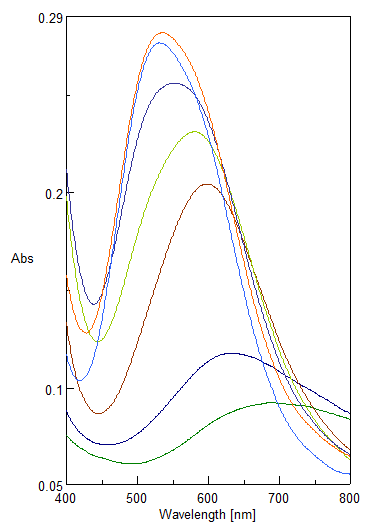

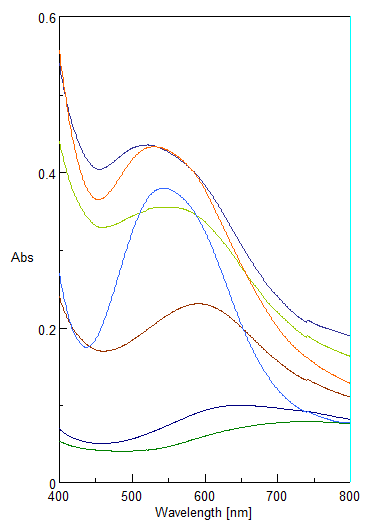


pH4

pH10

pH11

pH5

pH5

pH11

pH9

pH8

pH6

pH7

pH10

VEGFQ79G +Cu 2.4:1

(**a**)

VEGFI83G +Cu 0.8:1

VEGFI83G +Cu 1.6:1

VEGFI83G +Cu 2.4:1


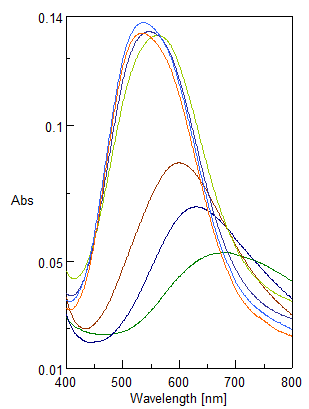


pH11

pH5


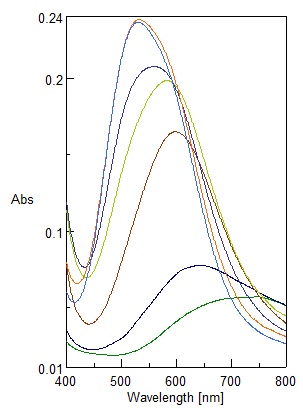

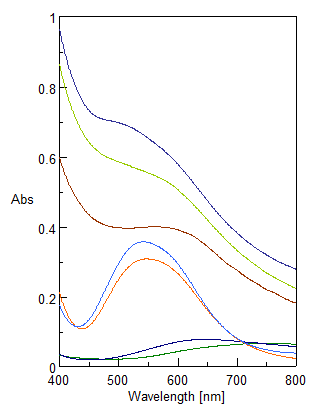


pH5

pH11

pH5

pH11

pH9

pH8

pH7

pH10

pH6

(**b**)

**Figure S7.** UV-Vis titrations of (**a**)VEGFQ79G and (**b**)VEGFI83G peptides at 1:0.8, 1:1.6, 1:2.4 ligand to metal molar ratio, in water solutions, 5<pH<11 range.

**Figure S8.** Far-UV CD comparison of VEGF73-101 and VEGFI83G (black); VEGF73-101/Cu(II) and VEGFI83G/Cu(II) in membrane (red).

| 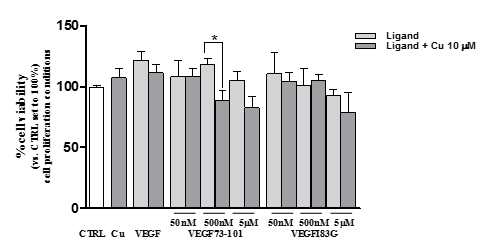 |  |
| --- | --- |
| (**a**) | (**b**) |


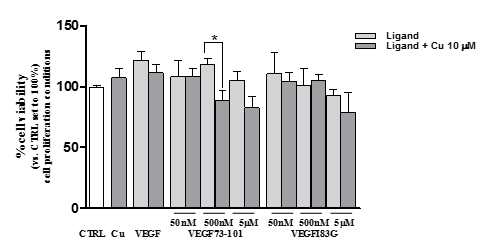


1. **(b)**

**Figure S9.** Evaluation of copper effects on VEGF165 (10 ng/mL), VEGF73-101 and VEGFI83G. (**a**) HUVEC cells (3 x 10^3^ cells/wells) were treated for 48 h in EMB2 basal medium with 0.2% FBS without growth factors. (**b**) Cells (1.5 x 10^3^ cells/wells) were treated for 48 h in EMB2 basal medium with 2% FBS with growth factors and without VEGF165. At the end, the cell viability was evaluated by MTS assay. Values are expressed as the percentage of viable cells with respect to untreated cells (CTRL). Data are the mean±SEM of three different experiments performed in triplicate. Statistically significant differences are indicated with *=p<0.05 vs. CTRL (One-Way ANOVA + Tukey’s test).

**Figure S10.** Effects of copper chelation on VEGF73-101 activity in HUVEC cells. Cells (1.5 × 10^3^ cells/wells) were treated for 24 h in EMB2 basal medium with 2% FBS with growth factors without VEGF165, in the absence or presence of BCS (50 µM). At the end, the cell viability was evaluated by MTS assay. Values are expressed as the percentage of viable cells with respect to untreated cells (CTRL). Data are the mean ± SEM of three different experiments performed in triplicate. Statistically significant differences are indicated with *=p<0.05 vs. CTRL (One-Way ANOVA + Tukey’s test).
